# Unilateral macular serpiginous-like choroiditis as the initial manifestation of presumed ocular tuberculosis

**DOI:** 10.1186/s40942-020-00272-7

**Published:** 2021-01-04

**Authors:** Raul N. G. Vianna, Vinicius Vanzan, Maria Luisa Gois da Fonsêca, Leonardo Cravo

**Affiliations:** Retina and Vitreous Unit, Department of Ophthalmology, Fluminense Federal University, Marques do Parana Avenue 303 Centro, Niterói, RJ 24033-900 Brazil

**Keywords:** Macular serpiginous-like choroiditis, Serpiginous choroiditis, Tubercular serpiginous–like choroiditis, Tuberculosis

## Abstract

**Background:**

Classic serpiginous choroiditis (SC) usually begins in the peripapillary area and spreads centrifugally, however, in some patients, the lesion can arise in the macular region. An association between lesions resembling classic SC and tuberculosis was recognized as a possibly distinct clinical entity and named as tuberculous serpiginous–like choroiditis. The differentiation of this tuberculous entity from SC is critical because the treatment of the former with immunosuppressive drugs leads to several potential adverse effects, and such treatment can have devastating consequences because of the worsening of a concomitant tuberculous infection.

**Case presentantion:**

A 31-year-old woman presented with unilateral decreased vision and a fundus examination consistent with macular serpiginous choroiditis. A non-reactor tuberculin skin test and normal thoracic CT scan ruled out tuberculosis. However, after 2 months of treatment with steroids and immunosuppressive drugs, the contralateral eye developed similar lesions, further raising the suspicions of ocular tuberculosis. We conducted QuantiFERON® TB Gold, which was positive; hence, antituberculous therapy was started on the patient. The lesions started healing within a few weeks. After 1 year of finishing the therapy, the lesions remained healed without any recurrence.

**Conclusions:**

Macular serpiginous-like choroiditis may be the initial presentation of presumed ocular tuberculosis. Nevertheless, the correct diagnosis of this entity can be challenging and delayed by the imprecise results from the currently available methods.

## Introduction

Serpiginous choroiditis (SC) is a rare, usually bilateral, and chronically recurring inflammatory disease that affects the inner choroid and the retinal pigment epithelium (RPE). The classic SC usually begins in the peripapillary area and spreads centrifugally, in a snake-like manner, over a few months or years [1]. However, in some patients, the lesion can arise in the macular region. This observation led Mansur et al. to call this particular entity as “macular SC” (MSC) [2].

An association between lesions resembling classic SC and tuberculosis (TB) was recognized as a possibly distinct clinical entity and named as tuberculous serpiginous–like choroiditis (TSC) [3]. The differentiation of this tuberculous entity from SC is critical because the treatment of the former with immunosuppressive drugs leads to several potential adverse effects, and such treatment can have devastating consequences because of the worsening of a concomitant tuberculous infection [4]. On the contrary, antiTB treatment may also be associated with significant adverse events, especially in older patients with SC.

The purpose of this paper is to report the case of a young, otherwise, healthy woman who presented with unilateral decreased visual acuity initially diagnosed as MSC. A non-reactor tuberculin skin test (TST) and normal thoracic CT scan ruled out TB. However, during the treatment with steroids and immunosuppressive drugs, the contralateral eye developed SC-like lesions, further raising the suspicions of ocular TB. We conducted QuantiFERON® TB Gold (QFT-G), which was positive; therefore, antituberculous therapy (ATT) was started on the patient. After 1 year of finishing the therapy, the SC-like inflammatory lesions were healed without any recurrence.

### Case report

**Fig. 1:**
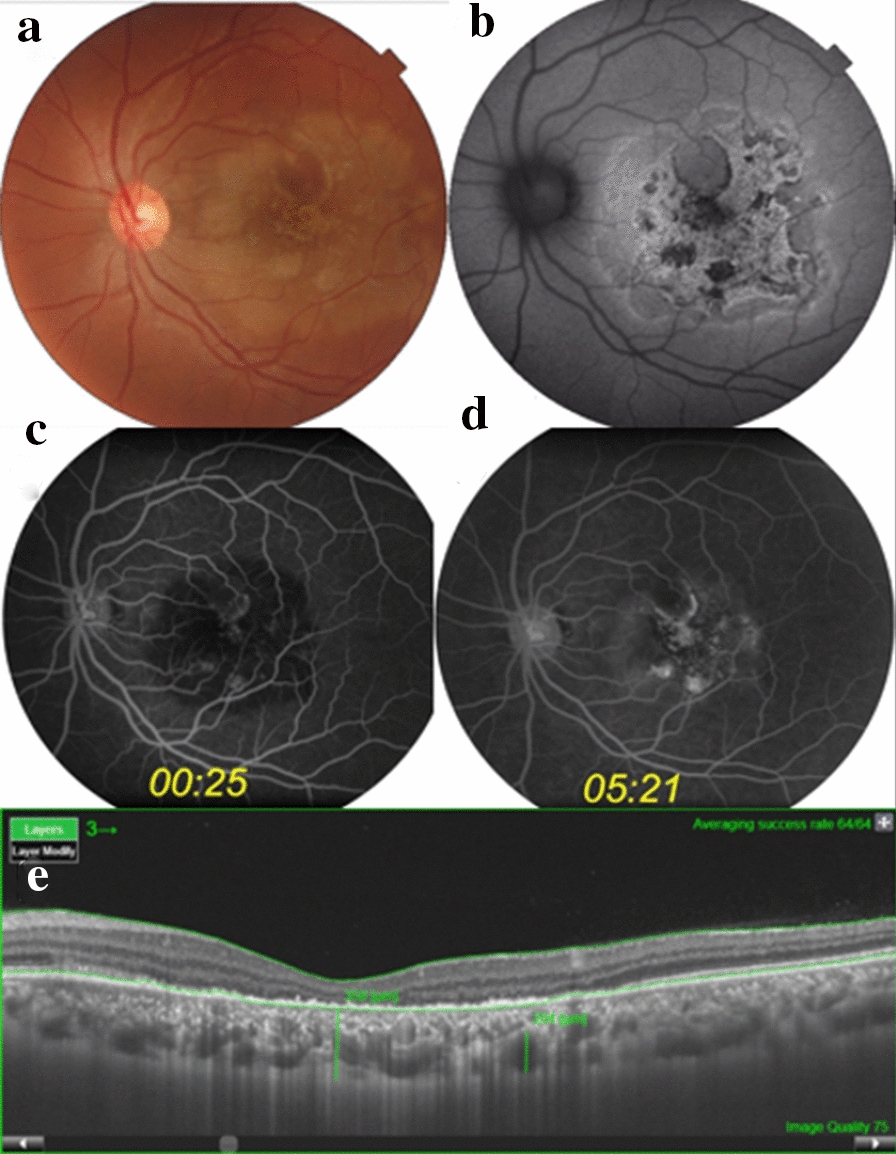
**a** Color photo of the left eye showing the yellow-white placoid lesion at the level of the RPE and outer retina, centered in the fovea. **b** The lesion is hyperautofluorescent at the acute stage. **c**, **d** Fluorescein angiography displays typical early hypofluorescence with late staining. **e** SS-OCT reveals a decreased foveal thickness and disruption of the outer retina. Observe the increased thickness of the choroid as well and the pachyvessels. (RPE, retinal pigment epithelium; SS-OCT, swept source optic coherence tomography)

A 31-year-old previously healthy woman had an acute loss of vision in the left eye (LE) since the last 12 days. Her corrected vision acuity was 20/20 in the right eye (RE) and 20/400 in the LE. The slit-lamp examination of the anterior segments was unremarkable. Intraocular tension was within the normal limits in both eyes. No cells were observed in the vitreous region of the LE. A pale, round, yellow-white placoid lesion at the level of the RPE and outer retina was observed inside the vascular arcades of the left macula (Fig. 1a). The optic disks and retinal vessels had a normal appearance.

The lesion showed a thin hyperautofluorescent border, as well as some round regions of hypoautofluorescence inside a hyperautofluorescent background (Fig. 1b). Fluorescein angiography revealed initial hypofluorescence with later hyperfluorescence of the whole lesion, mainly at the foveal and parafoveal regions probably because of RPE atrophy (Fig. 1c, d). The retinal vessels and the optic disks remained with normal fluorescence during all the phases of the examination. Indocyanine green angiography (ICGA) revealed a hypofluorescent plaque during all the phases. Swept-source optic coherence tomography (SS-OCT) displayed a decreased thickness of the fovea, many hyper-reflective dots at the level of the RPE, with a granular appearance, as well as the destruction of the external retina; however, it did not observe the ellipsoid layer and the external limiting membrane (Fig. 1e). The lesion appeared hyper-reflective at the outer retina but 9 × 9 OCT angiography visualized it much better at the choriocapillaris slab. Both superficial and deep plexus were apparently unaffected.

We conducted VDRL, FTA-abs, TST and IgM/IgG for toxoplasmosis, and chest CT, and found that these test results were normal (including a nonreactor TST). As the most prevalent infectious diseases commonly observed in South America were ruled out by laboratory and CT scans, the patient began treatment for MSC. Thereafter, pulsotherapy (methylprednisolone 1 g/day/3 days), oral corticosteroids (1 mg/Kg/day) and azathioprine (AZA) (3 mg/Kg/day) were started on the patient.

**Fig. 2:**
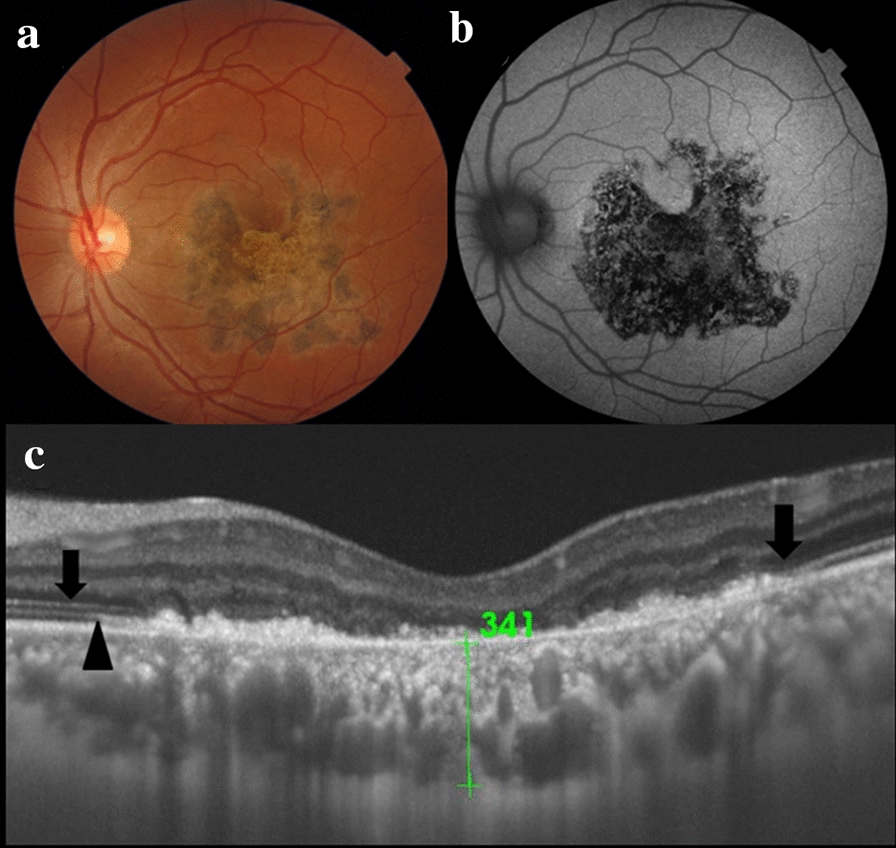
**a** The pigmented lesion after 2 months of treatment. **b** The lesion showed total hypoautofluorescence after healing. **c** SS-OCT revealed foveal degeneration but the external limiting membrane (arrows) and the ellipsoid layer (arrow head) could already be observed at the perifoveal region. (SS-OCT, swept source optic coherence tomography)

Within 2 months, the visual acuity was recovered to 20/63 in the patient. The lesion now appeared pigmented (Fig. 2a) and, totally, hypoautofluorescent (Fig. 2b). FA showed a well-demarcated lesion with hyperfluorescent borders and the round spots of RPE atrophy inside a hypofluorescent background. OCT now revealed foveal degeneration but the external limiting membrane and the ellipsoid layer could already be observed in the perifoveal region (Fig. 2c). She continued taking AZA (150 mg/day) and oral prednisone (20 mg/day). The patient was followed up for every month.

**Fig. 3:**
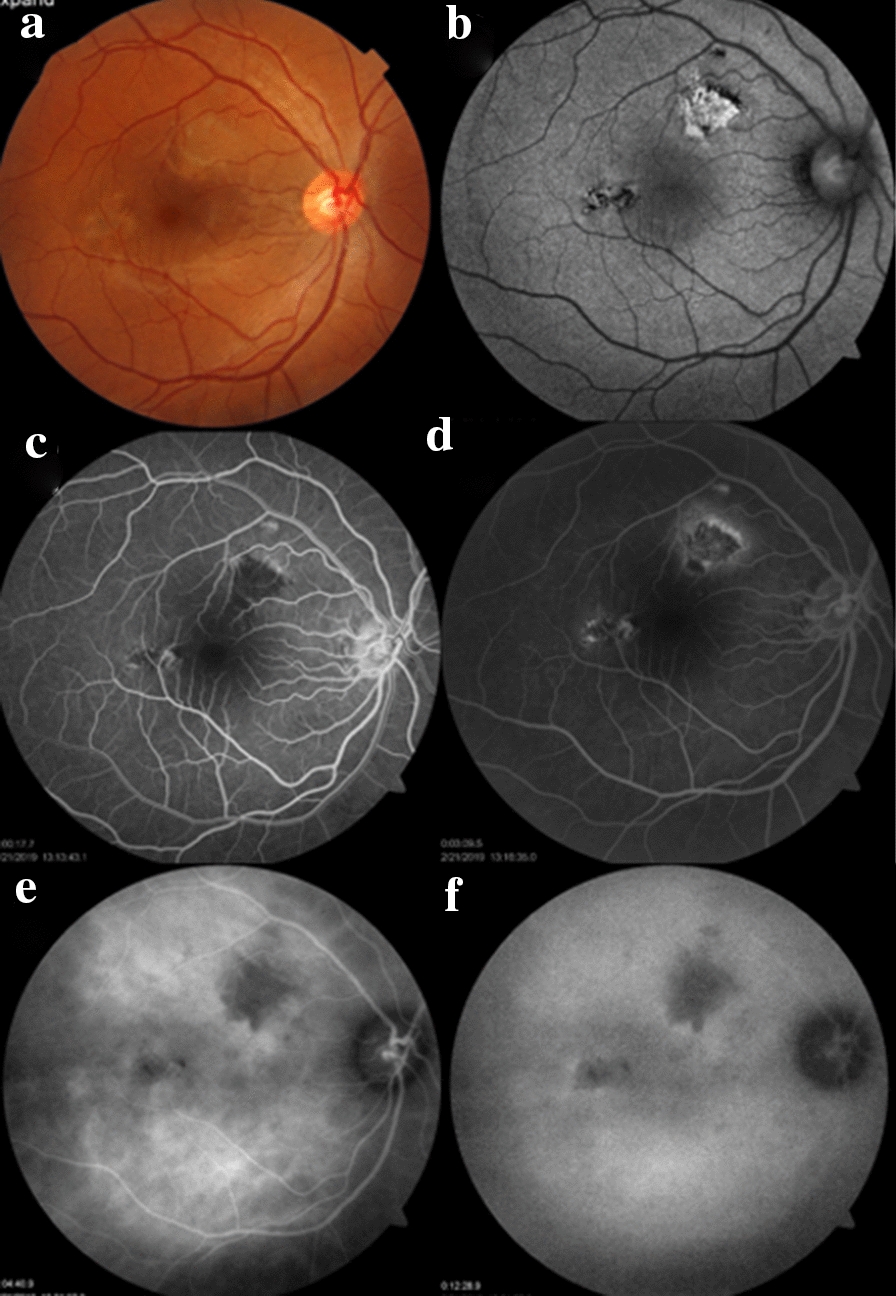
**a** Two deep creamy lesions in the macula of the right eye. **b** The superior lesion is hyperautofluorescent, but the other is hypoautofluorescent. **c** and **d** Fluorescein angiography shows the pattern of early hypofluorescence with late staining. Indocyanine green angiography in (**e**) early and (**f**) late phases revealed that the lesions were hypofluorescent during the whole examination

Two months after the above-described follow-up visit, the patient was asymptomatic, but we detected two deep creamy lesions in the macular area of the RE (Fig. 3a). The superior and larger lesions showed hyperautofluorescence, which is typically observed in the active disease. The smaller one showed a mixed pattern of autofluorescence (Fig. 3b). Both lesions were hypofluorescent with late hyperfluorescence on FA (Fig. 3c, d), and hypofluorescent in all the phases of ICGA (Fig. 3e, f).

**Fig. 4:**
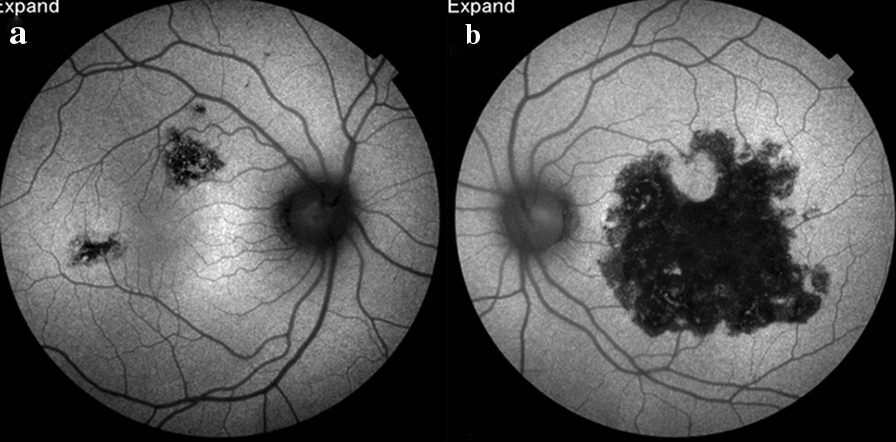
**a** Right and (**b**) left eyes 1 year after finishing ATT showing total hypoautofluorescence of the healed lesions

Although TB has been ruled out by a non-reactor TST and thoracic CT scan, we conducted the interferon-gamma release assay (IGRA), via QFT-G methodology, which was positive. We then initiated ATT (isoniazid, rifampin, ethambutol, and pyrazinamide) for 9 months. AZA was immediately withdrawn. A maintenance dose of corticosteroids (60 mg/day, slowly tapered within 3 months) was continued until the lesions showed total hypoautofluorescence. One year after finishing ATT, the lesions were stable with no recurrence in both the eyes (Fig. 4a–d), and the visual acuity was 20/20 in the RE and remained at 20/63 in the LE.

## Discussion

TSC typically affects young- to middle-aged adults from the endemic areas of TB, with a male preponderance [5]. Bilateral involvement is a common feature of the disease, which may present as the active lesions in both the eyes simultaneously, alternate in one eye followed by the involvement of the other eye, or active in one eye with inactive scars in the other eye [5]. In the larger series so far, Bansal et al. reported that two distinct patterns may be observed in this entity. The more common form (95% of the cases) with multifocal lesions of active choroiditis that were discrete and noncontiguous, to begin with, and then progressed relentlessly to a diffuse, contiguous variety, thereby acquiring an active advancing edge resembling SC. The other one is a rare form (5% of the cases) that presents with a diffuse plaque-like choroiditis showing amoeboid spread [5]. Our patient can be classified in this latter form. However, different from those patients described by Basal et al. our case presented unilaterally and without detectable vitreous cells. These features, as well as the non-reactor TST, led us to the initial diagnosis of “idiopathic” MSC [2].

In fact, the diagnosis of intraocular TB is often problematic due to a wide spectrum of presentations and it is impractical to conduct a biopsy for culture and direct histopathological examination to provide definitive proof of ocular infection. In nearly all reported cases, the diagnosis of ocular TB was only presumptive [6]. In most studies, the diagnostic criteria for presumed ocular TB were: residence or migration from areas endemic in TB, history of contact with patients with TB infection, presence of suggestive ocular findings, exclusion of other known causes of uveitis, corroborative evidence, such as a positive TST, positive IGRAs, and a positive response to conventional ATT without recurrence [6].

Regarding the laboratory examinations, both TST and QFT seem to be equivalent to the diagnosis of ocular TB. Kurup et al. reported no demonstrable advantage of QFT-G assay over the TST in detecting latent TB infection in patients with granulomatous uveitis [7]. However, TST has the following disadvantages when it is compared with QFT-G: it is subjective, can result in false-positive in previous patients vaccinated with Bacilli Calmette-Guerin (BCG), as well as can be false-negative in immunocompromised patients. In fact, in our case, we initially ruled out TB based on a non-reactor TST. QFT-G is an interferon-gamma release assay, commonly known as an IGRA, which is an alternative to TST. Unlike TST, QFT-G is a controlled laboratory test that is unaffected by previous BCG vaccination. QFT-G is highly specific and sensitive; a positive result is strongly predictive of true infection with *Mycobacterium tuberculosis* [8]. However, like the TST and other IGRAs, QFT-G cannot distinguish between active TB disease and latent infection and is intended to be used for risk assessment, images, and other medical and diagnostic evaluations. Like any diagnostic aid, QFT-G cannot replace clinical judgment. We have considered the positive QFT-G presented by our patient that was associated with the retinal SC-like lesions and supported our diagnosis of presumed ocular TB. Moreover, our region is a moderate endemic area for TB, which enhanced the value of a positive QFT-G for the diagnosis. In India, where TB is highly prevalent, Babu et al. reported a significant association of serpiginous-like choroiditis and positive QFT-G [9] Mackensen et al. in their study from a low TB endemic country have also reported a 52% QFT-G positivity in serpiginous-like choroiditis subset of tubercular uveitis [10]. This association strengthens the need to rule out TB in all patients with serpiginous-like choroiditis, regardless of whether the region is endemic or not. Another important fact that can support the diagnosis of presumed ocular TB in our patient is that the lesions did not recur 1 year after finishing ATT. Intriguingly, in one study, 50 patients presented with multifocal choroiditis due to presumed TB and were treated with ATT without any concomitant use of systemic corticosteroids [11]. All patients had a favorable response, and no recurrence was observed. These findings may indicate that ocular manifestations in these patients were probably because of direct mycobacterial invasion. Therefore, it is plausible to hypothesize that this could also have been the mechanism of infection in our patient.

The failure to consider TB in the differential diagnosis of intraocular inflammation may have catastrophic consequences because the immunosuppressive agents often used to manage the intraocular inflammation may be fatal for both vision and life in patients with active TB disease [12]. In our case, the MSC-like lesion of the RE healed promptly a few weeks after the anti-inflammatory therapy, but 2 months later new lesions appeared in the contralateral eye. We immediately removed the AZA, but a maintenance dose of corticosteroids was continued until the lesions of both eyes were healed. In fact, systemic steroids, in addition to ATT, are sometimes considered for patients with persistent ocular inflammation or retinal vasculitis, but no clinical trials have proven their efficacy.

## Conclusion

We believe that our report can contribute to the literature by presenting a rare form of SC-like lesion in a patient with a difficult diagnosis. Moreover, the current methods for the detection of ocular TB must be cautiously interpreted. We also stress that TB must be ruled out in all forms of presentation of SC.
